# A Three-Decade Overview of Cadmium and Lead in Placentas of Postpartum Women: A Review of Evidence from Croatia (1990s–2019)

**DOI:** 10.3390/life16020343

**Published:** 2026-02-16

**Authors:** Tatjana Orct, Ankica Sekovanić, Zorana Kljaković-Gašpić

**Affiliations:** Institute for Medical Research and Occupational Health, 10000 Zagreb, Croatia; torct@imi.hr (T.O.); zorana.kljakovic-gaspic@imi.hr (Z.K.-G.)

**Keywords:** toxic metals, placenta, prenatal exposure, mother-newborn pairs, smoking, environmental exposure

## Abstract

Toxic heavy metals, including cadmium (Cd) and lead (Pb), can build up in placental tissue or pass through the placental barrier, potentially harming fetal development. Therefore, the placenta can serve as a useful tool for assessing prenatal exposure to these harmful substances. Over the past several decades, Croatia has implemented a range of environmental and public health measures aimed at reducing exposure to Cd and Pb, including ratification of the World Health Organization Framework Convention on Tobacco Control (FCTC), ban on smoking in public places, intensified health education campaigns, and the complete phase-out of leaded gasoline in 2006. As a result, smoking prevalence among women and Pb levels in ambient air have declined substantially. This study reviews and analyzes existing literature on Cd and Pb levels in placental tissue of women in Zagreb, Croatia, in order to evaluate the effectiveness of these health and environmental policies and to identify persistent or emerging risks associated with toxic metal exposure during pregnancy by comparing placental Cd and Pb levels between smokers and nonsmokers across several time periods.

## 1. Introduction

Cadmium (Cd) and lead (Pb) are well-known environmental and industrial toxicants that pose serious health risks, especially to vulnerable groups such as pregnant women and developing fetuses. In the general population, cigarette smoking is one of the most significant non-occupational sources of exposure to toxic elements, particularly Cd [1,2], whereas food is the primary source among nonsmokers [3]. In the case of Pb, historical exposure was largely attributed to the use of leaded gasoline, with ambient air serving as the main exposure route before this fuel was banned or phased out in many countries, including Croatia. Following the transition to lead-free gasoline, cigarette smoking has become the primary source of Pb exposure in smokers.

Despite the World Health Organization’s recommendation that pregnant women should not smoke [4], many do not cease tobacco use entirely and instead only reduce consumption during pregnancy. According to a recent meta-analysis study, 52.9% (95% CI 45.6–60.3) of women who smoked prior to pregnancy continue to smoke during pregnancy [5]. A multinational, web-based study [6] involving pregnant women from 15 European countries, including Croatia, reported that 18.9% of Croatian pregnant women continue to smoke during pregnancy. In contrast, the prevalence of smoking during pregnancy is below 7% in the United Kingdom, Norway, and Slovenia, and below 5% in Austria and Iceland [6]. Smoking during pregnancy endangers both the unborn child and the mother. Numerous studies have shown that smoking during pregnancy increases the risks of miscarriage, low birth weight, preterm delivery, stillbirth, and sudden infant death syndrome [7,8]. Importantly, cigarette smoke is not only a source of Cd and Pb but a complex mixture of various hazardous substances, including nicotine, carbon monoxide, polycyclic aromatic hydrocarbons, and other metals. These substances may exert direct adverse effects through transplacental transfer of toxins, or indirect effects by altering the vascular structure of the placenta, thereby affecting blood flow in the umbilical arteries and reducing nutrient transfer across the placenta [9,10]. Therefore, although the present research primarily focuses on Cd and Pb as important exposure markers, differences observed between smokers and nonsmokers likely reflect the combined influence of multiple smoke-related toxicants.

The placenta is a unique organ that links the mother and fetus into a single, functional maternal–placental–fetal unit. Within the scientific community, it is widely recognized as a readily accessible tissue for assessing metal exposure in both mother and fetus during the early stages of development [11,12,13,14]. Nevertheless, when using placental samples to assess metal exposure, it is crucial to carefully collect a representative sample, as the placenta is a large and structurally heterogeneous organ containing diverse cells of maternal and embryonic/fetal origin, and includes remnants of maternal and fetal blood [15]. In Croatia, research on placental exposure to Cd and Pb was initiated in the 1990s and has been carried out continuously since the early 2000s by researchers from the Institute for Medical Research and Occupational Health (IMROH). An integrated overview of maternal and fetal exposure over several decades is provided by the prolonged nature of this research, as well as consistent methodological and analytical approaches, which allow for a trustworthy assessment of temporal patterns in placental Cd and Pb levels. The present study integrates all previously published data on Cd and Pb concentrations in placentas of women who gave birth at Zagreb maternity hospitals, with the aim of assessing temporal variations in placental concentrations of these harmful metals. Particular attention is given to differences between smokers and nonsmokers, as well as to the potential impact of air pollution and smoking-related legislation on exposure patterns. To minimize geographical variability and the influence of region-specific dietary habits, only pregnant women from Zagreb and the surrounding area were included. By synthesizing these data using a consistent methodological framework, this work offers a three-decade overview of placental Cd and Pb exposure in relation to significant socioeconomic and regulatory developments, extending earlier Croatian research that was mostly limited to single time points or brief study periods.

## 2. Materials and Methods

### 2.1. Data Sets

The data set used in this study was assembled through a search strategy across major scientific databases (Web of Science, PubMed, and Scopus) using the keywords: *placenta*, *cadmium*, *lead*, and *Croatia*. We included data only for pregnant women from Zagreb and the surrounding area who gave birth in Zagreb maternity hospitals and had not been previously occupationally exposed to toxic metals, specifically Cd and Pb. Their exposure thus reflects everyday unavoidable environmental sources and lifestyle-related factors (e.g., smoking, diet). Although one of the earlier studies also involved pregnant women from Zadar and its surroundings [13] and examined geographical differences in placental Cd levels of nonsmokers, only data for subjects from Zagreb were extracted for the present analysis. This restriction is justified by the Croatian Geochemical Atlas, which documents regional differences in soil Cd levels [16] that may affect Cd levels in food, and, ultimately, in the body. The final dataset comprises manually extracted summary data from published papers that reported Cd and Pb concentrations in placentas of smoking and nonsmoking women across three periods: the 1990s (two papers [17,18]), 2008–2010 (three papers [13,19,20], and 2017–2019 (three papers [21,22,23]). An exception applies to Pb, for which placental data for smokers and nonsmokers in the 2008–2010 period were available only in one publication [20], while the other two studies from this period [13,19] did not report Pb concentrations. Subjects were classified as smokers or nonsmokers based on self-reported data obtained through questionnaires, as described in the original studies. All included studies involved healthy women who had vaginal births at term. Data are presented as reported in the source publications: as mean ± standard deviation in the first paper from the 1990s [17], and as median with interquartile range in the remaining papers, except for Piasek et al. [19], where results are given as median and range. All studies from which the data were obtained received appropriate ethical approval and were conducted in accordance with the Declaration of Helsinki.

### 2.2. Sampling and Analysis Methods

One study from the 1990s [17] reported that immediately after delivery, the placenta was weighed, placed in a plastic bag, and transferred to the laboratory for storage at −20 °C. Three subsamples were taken for metal analysis: one from the central part, avoiding the umbilical cord insertion, and two from between the central and the peripheral region within 3 cm of the outer placental margin. Another study from the 1990s [18] also reported that full-term placentas were placed in a plastic bag, transferred to the laboratory, and stored at −20 °C. After thawing, placentas were washed in physiological saline solution (0.9% NaCl), blotted on filter paper, trimmed of umbilical cord and extraembryonic membranes, and weighed. The placenta was then cross-cut through its entire thickness, and two subsamples were taken, excluding the chorionic plate: one from the central region (avoiding the insertion point) and one from the region between the centre and periphery, approximately 3 cm from the disc margin. Among the studies from 2008–2010, two did not provide a detailed description of sample collection [13,19], while one study [20] reported a procedure similar to Stasenko et al. [18], with the additional detail that 1–2 mm thick slices were removed from both the maternal and fetal sides, washed with ultraclean water, and dried. A slightly different sampling approach was reported in the studies from 2017–2019 [21,22,23]. In these studies, placentas were not frozen after delivery; instead, they were transported to the laboratory and processed fresh. After blotting on filter paper, trimming the umbilical cord and extraembryonic membranes, and weighing, full-thickness sections were collected: one from the midline of the placental disc (avoiding the insertion site) and two from the intermediate region between the central and peripheral parts, excluding the outermost 3 cm of the disc margin. From these samples, only trophoblastic tissue was retained by removing the decidua basalis and chorionic membrane plate, and 1–2 g of fresh tissue was stored at −80 °C [21,22,23].

The included studies also slightly differed in sample preparation procedures prior to Cd and Pb determination. In the 1990s study [17], 2–3 g cubes of fresh placental tissue, one from the central paraumbilical section and two from the peripheral section, were rinsed with saline, blotted on filter paper, weighed, and dried at 105 °C to constant mass. Samples were then dray-ashed in quartz glass crucibles at 450 °C in a muffle furnace and subsequently dissolved in 2% nitric acid. Detailed sample preparation methods prior to metals analysis were not reported in Stasenko et al. [18]. For the 2008–2010 period, one study did not describe sample preparation [13], one reported only acid digestion in an UltraCLAVE high-pressure microwave system [19], while one provided more detailed preparation procedure [20]: 1–2 g of tissue was digested with nitric acid and water (2:1) in an UltraCLAVE high-pressure microwave system, and the digests were then brought to a final mass of 8 g with ultrapure water. For the 2017–2019 studies, sample preparation was not described in great detail. Two papers reported acid digestion of 0.5 g [21] or approximately 1 g [22] of tissue using an UltraCLAVE microwave digestion system. Additional details were provided in Piasek et al. [23], where samples were digested with nitric acid and ultrapure water (1:1) in an UltraCLAVE high-pressure microwave system and then brought to a final mass of 5 g with ultrapure water.

For the metal determination step, electrothermal atomic absorption spectrometry (ET-AAS) with a graphite furnace atomizer and deuterium background correction was employed in the 1990s studies [17,18], whereas inductively coupled plasma–mass spectrometry (ICP–MS) was used in the studies published from 2008 onward [13,19,20,21,22,23]. All studies reported placental average concentrations calculated as the mean of the peripheral and central subsample values, because Piasek et al. [17] reported no differences in Cd and Pb concentrations between sampling sites within the placenta, consistent with findings reported elsewhere [18,20].

### 2.3. Data Analysis

Data analysis was performed using the R software package, version 2025.09.2 (R Foundation for Statistical Computing, Vienna, Austria) and TIBCO Statistica™, version 14.0.0.15 (TIBCO Software, Inc., Santa Clara, CA, USA). Annual PM_10_-based concentrations of Pb and Cd in ambient air in Zagreb for the period 2017–2019 were obtained from the Ministry of Environmental Protection and Green Transition, specifically from the Zagreb-1 and Zagreb-3 monitoring stations, covering the interval from 1 January 2017 to 31 December 2019 [24]. Annual concentrations of Cd and Pb in PM_10_ for 2008–2010 were taken from the document “Air Quality Assessment” of the Croatian Meteorological and Hydrological Service, Zagreb [25], and from Vađić et al. [26] for the monitoring station Zagreb-1. Cd concentrations in PM_10_ were not available for the 1990s. For the same period, annual Pb concentrations in total suspended particulate matter were obtained from the document “Report on the air quality in the Republic of Croatia” [27], as average concentrations across four monitoring locations for the years 1991–1999. In the present study, all ambient Cd and Pb values are presented as the average annual concentrations in Zagreb air. Statistical analyses were limited to descriptive assessment of temporal trends. No inferential statistical testing was undertaken because the analysis relied on published, study-level summary data from multiple cross-sectional studies that reported results using heterogeneous descriptive metrics (mean ± SD, median with interquartile range, or median with range; Table 1). This heterogeneity precluded valid pooling, consistent variance estimation, and formal hypothesis testing across periods. Comparability of placental Cd and Pb data across study periods is supported by the fact that all measurements were performed within the laboratories of the same research institution using established analytical methods, consistent method verification, and quality assurance/quality control procedures.

## 3. Results and Discussion

Data on Cd and Pb levels in the placental tissue of women from Zagreb, as reported in the literature included in this study, are summarized in Table 1. As evident from the table, only a limited number of studies have been conducted in this sensitive population group. For the first two studies, the original publications did not specify the exact years of sample collection; based on publication dates and contextual information, sampling is assumed to have occurred in the 1990s, and these data are referred to accordingly in Table 1. Three studies were conducted between 2008 and 2010, and three between 2017 and 2019. Of the three studies conducted between 2008 and 2010, two did not measure Pb in the placenta and reported only Cd. Beyond the studies included here, one additional investigation in the Croatian population reported placental Cd concentrations in both normal pregnancies and those with intrauterine growth restriction [28]. However, this study was excluded from the analysis because it was conducted in Osijek, not in Zagreb, and focused on pregnancies with fetal growth disorders—a population differing from the general obstetric population, thus limiting comparability with the target population. To maintain geographic and population comparability and to better characterize temporal trends in placental Cd and Pb levels, we restricted the dataset to studies conducted in the maternity wards in Zagreb. While this approach limits the generalizability of the findings to other regions of Croatia, it improves internal consistency and reduces confounding caused by region-specific differences in environmental contamination and diet patterns. In all included studies, smoking status was identified as the main source of exposure to Cd and Pb and served as the basis for participant categorization.

**Figure 1:**
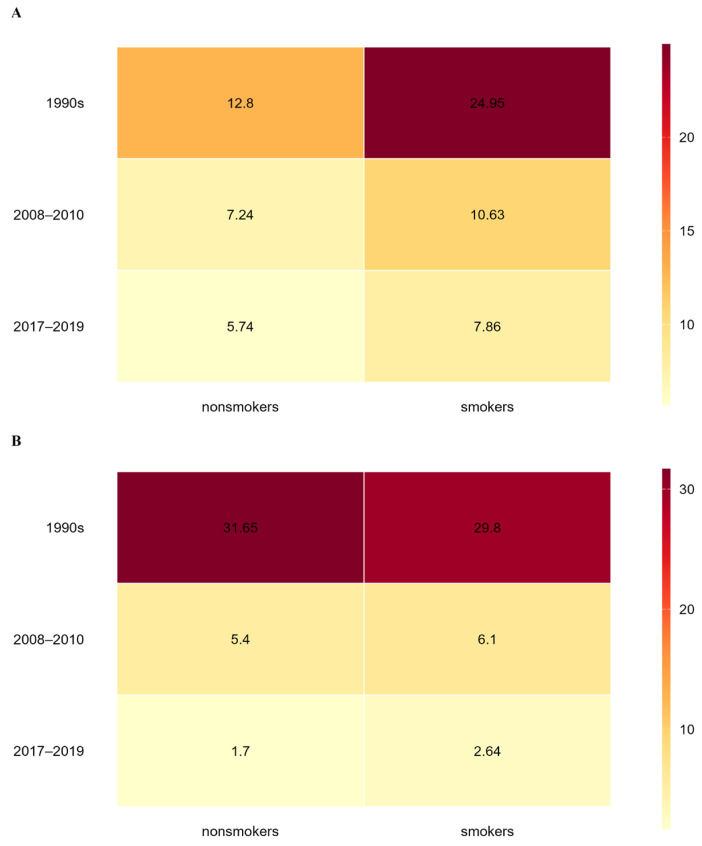
Heat maps illustrating temporal trends in average placental concentrations of (**A**) cadmium and (**B**) lead in nonsmokers and smokers over the period 1990–2019. Darker shades indicate higher metal levels in the placenta, while lighter shades represent lower levels.

Taken together, the compiled data indicate a clear long-term decline in placental Cd and Pb levels among women from Zagreb over the three-decade observation period. Although formal statistical testing of differences between study periods or population groups was not feasible due to heterogeneous reporting of summary statistics and the absence of individual-level data, the consistency in the direction of change across independent studies supports the presence of a genuine temporal trend. Figure 1 presents a heat map that facilitates integrated comparison of Cd (Figure 1A) and Pb (Figure 1B) exposure gradients in nonsmokers and smokers. Lighter natural/yellow shades denote lower levels of toxic metals in the placenta, while darker red shades represent Cd and Pb concentrations exceeding 20 ng/g wet weight. Women who gave birth in the 1990s had the highest placental metal burdens, with overall mean concentrations of 18.9 ± 8.0 ng/g wet weight for Cd and 30.7 ± 17.6 ng/g wet weight for Pb. By contrast, women recruited between 2017 and 2019 had the lowest levels, with overall mean values of 6.8 ± 1.2 ng/g wet weight for Cd and 2.2 ± 0.57 ng/g wet weight for Pb. As illustrated for Cd in Figure 1A, placental Cd concentrations in the 1990s were roughly twofold higher in smokers than in nonsmokers. This difference diminished in 2008–2010 by 25%, when smokers exhibited Cd levels about 1.5 times those of nonsmokers. A comparable pattern persisted in the 2017–2019 period, with Cd concentrations in smokers remaining approximately 1.4-fold higher than in nonsmokers. For Pb, placental concentrations were nearly identical in smokers and nonsmokers in the 1990s. In 2008–2010, smokers had Pb levels 1.1 times higher than nonsmokers, and this difference increased to 1.5-fold in 2017–2019. When analyzing temporal changes, placental Cd concentrations in smokers decreased by 57% in 2008–2010 compared with the 1990s, while the corresponding decrease in nonsmokers was 43%. Pb levels in placentas decreased by 79% in smokers and 83% in nonsmokers over the same time period. While Pb levels showed a more significant drop of 57% in smokers and 68% in nonsmokers in the subsequent period (2017–2019), Cd levels continued to decline, but less sharply (by 26% in smokers and 21% in nonsmokers) compared with 2008–2010.

**Figure 2:**
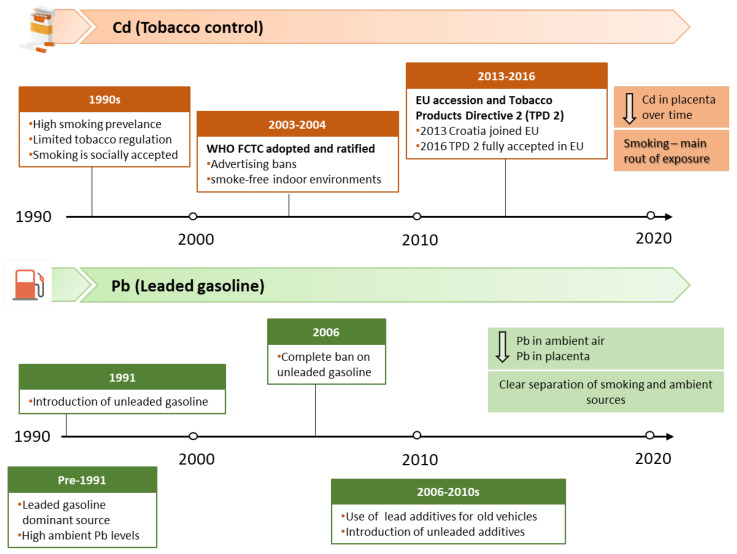
Summary of key regulatory milestones updated in Croatia from 1990 to 2019.

This observed downward trend in placental Cd and Pb concentrations over time likely reflects multiple concurrent factors, but they are most plausibly aligned with the stepwise implementation of key exposure reduction measures in Croatia from 1990 to 2019 (Figure 2). For Cd, the decline is most plausibly linked to the progressive strengthening of tobacco-control strategies, given that smoking is a key non-occupational contributor to Cd body burden, alongside broader socioeconomic changes, such as shifts in the educational structure of the population. The highest placental Cd levels in the 1990s occurred largely before comprehensive national smoke-free regulation. The decline evident by 2008–2010, followed by a further reduction in 2017–2019, coincides with the successive implementation of international and national tobacco-control frameworks, including a key milestone: adoption of the Framework Convention on Tobacco Control (FCTC) by the World Health Organization (WHO) in 2003, which aims to protect present and future generations from the harmful health, social, environmental, and economic consequences of tobacco use and exposure to tobacco smoke, thereby reducing tobacco-related morbidity and mortality [29]. Croatia ratified the FCTC in June 2004 and subsequently introduced a series of measures and regulations, including restrictions on indoor smoking, bans on tobacco sponsorship, promotion and advertising, and actions against tobacco smuggling. Together, these policy measures plausibly contributed to a gradual decline in smoking prevalence among women and reduced tobacco-related exposure over time, with corresponding reductions in placental Cd concentration, particularly among smokers.

A cross-sectional study conducted in 1995–1997, before the ratification of FCTC, reported that 26.6% of women in Croatia between the ages of 18 and 65 were daily smokers [30]. Padjen et al. documented a decrease in smoking prevalence among women from 31.6% in 1994–1998 to 26.6% in 1999–2001, and 21.7% in 2002–2005 [31]. Similar findings were obtained in the Tobacco Questions for Surveys (TQS) study conducted in 2014–2015 in individuals aged ≥15 years, where 27.1% of women were smokers (23.4% daily, 3.7% occasional) [32]. Subsequent data from 2016 showed a further reduction, with 20.5% of women reporting that they smoked [33]. In addition, during the 1990s, smoking was widely perceived as a sign of maturity and independence, an image that was carefully promoted by the tobacco industry [34], whereas in later years, social norms shifted toward greater disapproval of smoking. Concurrently, substantial changes occurred in the educational structure of the population over the 1990–2019 period. According to the 1991 census of the Croatian Bureau of Statistics, only 9.5% of adults had a university degree, 36.5% had completed secondary education, and 54.0% had only primary education. By 2001, the percentage of the population with a university degree rose to 12.0%, while the percentages with only primary and secondary education fell to 40.6% and 47.4%, respectively. Higher education continued to rise, reaching 16.4% in 2011 and 24.1% in 2021 [35]. Higher education is typically linked to decreased smoking prevalence, better health literacy, and a greater understanding of the negative impacts of smoking. The observed decrease in placental Cd levels was probably caused by this factor, as well as extensive tobacco-control measures, such as public smoking bans and increased health education programs. At the same time, the tobacco industry has adapted its marketing strategies. A systematic web-based review of documents released under the Master Settlement Agreement showed that the tobacco industry has conducted extensive research on women’s needs and preferences regarding tobacco products [36], leading to the development and promotion of flavoured cigarettes and other tobacco products. More recently, flavoured heated tobacco products with appealing aromas have emerged as an additional concern. Surveillance by the European Commission revealed a sharp increase in sales of heated tobacco products between 2018 and 2020, with an increase of about 2009% across all EU Member States and more than 999% in Croatia [37,38]. These trends may present new challenges for tobacco control and could affect future exposure to Cd, Pb, and other toxicants. Therefore, future studies should also focus on investigating the potential toxic effects of modern tobacco products. Furthermore, it should be noted that the most recent placental Cd data included in this analysis are from 2017–2019, i.e., after Croatia’s accession to the European Union in 2013 and after the EU Tobacco Products Directive 2 (2014/40/EU) [38] became applicable in May 2016. At present, no published placental biomonitoring data for Cd are available for this population group beyond that period. This highlights a significant knowledge gap and underscores the need for new placental biomonitoring studies to assess current exposure patterns under evolving tobacco products and regulatory frameworks.

**Figure 3:**
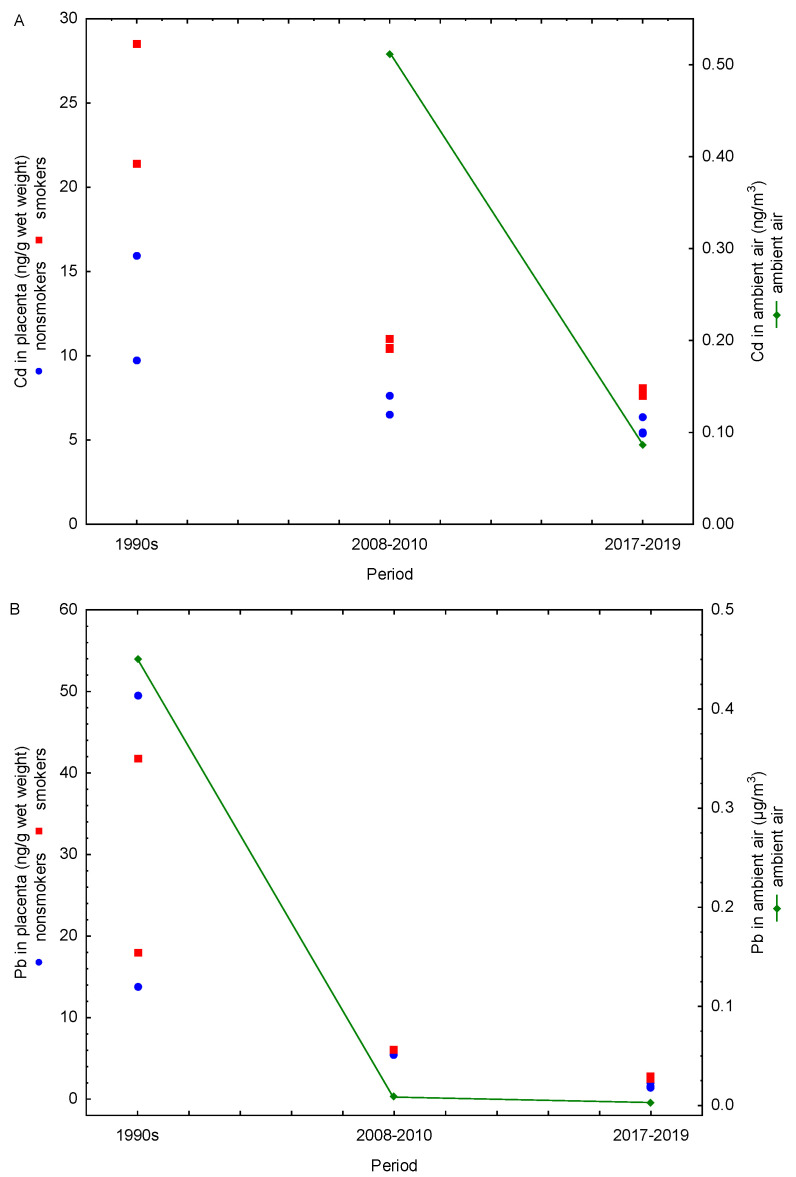
Temporal trends in annual (**A**) cadmium and (**B**) lead concentrations (1990–2019) in human placentas of smokers (red points) and nonsmokers (blue points), alongside their corresponding levels in ambient air (green line).

We also compared the amounts of Cd and Pb in the placenta with ambient air levels of these metals measured during the corresponding periods (Figure 3). Unfortunately, Cd concentrations in ambient air were not monitored during the 1990s; systematic monitoring of Cd in particulate matter with an aerodynamic diameter of ≤10 µm (PM_10_) started in 2006 [26], which enabled us to compare placental and ambient values only from this point onwards. Coal combustion, household waste incineration, and metallurgical operations, including ore mining and processing, are the primary sources of Cd in ambient air. It is subject to regular monitoring because it is classified as a Group 1 carcinogen, with well-documented evidence linking occupational Cd exposure and lung cancer. Although overall Cd levels in ambient air were higher in 2008–2010 than in 2017–2019, long-term data on average Cd levels for Croatia since the onset of monitoring show no clear upward or downward trend; instead, they exhibit year-to-year fluctuations over the monitoring period [24,26]. In contrast, as previously discussed in detail, smoking contributes much more to Cd inhalation than ambient air. Monitoring of Pb in total suspended particles in Zagreb started in 1991, while the measurement of Pb concentrations in PM_10_ particles began in 1999. The available data indicate that Pb concentrations in Zagreb ambient air dropped by 90% in 2008–2010 and by 99% in 2017–2019 compared with 1990 levels (Figure 3B). Historically, the primary source of lead in ambient air was leaded gasoline (containing alkyl-lead additives), introduced in the 1920s and used globally through the 1970s and early 1980s. As accumulating evidence identified leaded gasoline as a major threat to public health, many countries began to adopt stepwise measures to phase out and ultimately ban its use. In Croatia, lead-free gasoline was introduced in 1991, and its use increased by 5–10% annually until it represented 70% of total gasoline consumption in 2000 [39]. A 13-year Pb monitoring program in Zagreb demonstrated a pronounced decline in Pb levels in PM_10_ particles during 1999–2011, driven by the progressive reduction and final complete ban on leaded gasoline sales beginning on 1 January 2006 [26] (Figure 2). In the following years, vehicles without catalysts that previously used leaded gasoline transitioned to unleaded fuel with the addition of commercially available substitute additives. Demand for such solutions gradually decreased as older vehicles became obsolete. The substantial decline in ambient air Pb across study periods is also reflected in placental Pb levels, which exhibited a similar downward trend (Figure 3B), suggesting a reduced maternal and fetal exposure over time. This pronounced drop across study periods is consistent with the phased reduction of lead-related environmental sources, most notably the phase-out and subsequent ban of leaded gasoline. Accordingly, the high placental Pb levels observed in the 1990s correspond to a period when leaded gasoline was still widely used, whereas the significant declines in 2008–2010 and 2017–2019 coincide with the post-ban period and the gradual renewal of the vehicle fleet (Figure 2). In the 1990s, historically elevated Pb concentrations in ambient air resulted in similarly high placental Pb levels in both smokers and nonsmokers, as evidenced by the absence of significant differences between these groups [17]. In contrast, during 2008–2010 and 2017–2019, following the sharp decline in ambient air Pb, clear differences in placental Pb levels between smokers and nonsmokers became apparent [20,22,23]. These findings suggest that, in the 1990s, ambient air was the predominant source of human Pb exposure, effectively masking the contribution of cigarette smoke—particularly in pregnant women, as suggested by the comparable placental Pb levels in nonsmokers—and complicating the evaluation of smoke-related Pb effects on the placental and fetal health. After 2006, air Pb levels reportedly remained below 0.01 μg/m^3^, with an additional 65% reduction between 2008–2010 and 2017–2019 (0.0085 vs. 0.003 μg/m^3^). This decline is closely mirrored by decreases in placental Pb levels over the same period (57% in smokers and 68% in nonsmokers). If air Pb levels in the future remain low and stable, future studies may be better positioned to isolate and quantify the contribution of cigarette smoke and assess more accurately the health effects of cigarette smoke exposure on both mothers and fetuses.

Observed temporal patterns revealed distinct dominant exposure pathways for Cd and Pb in the monitored population. Across all study periods, placental Cd levels remained consistently higher in smokers than in nonsmokers, showing that cigarette smoking remains a persistent primary source of Cd exposure. Although Cd levels declined substantially from the 1990s onward, the rate of decrease appears to have slowed in recent years, suggesting that effect of tobacco control measures and reduction of smoking rates might have reached a plateau and that further reductions related to this source may be limited, with placental Cd concentrations potentially stabilizing near present levels. Conversely, placental Pb levels did not significantly differ between smokers and nonsmokers in the 1990s, indicating that the ambient air pollution, primarily driven by leaded gasoline, was the dominant source of exposure at that time. Following the phase out and subsequent ban of leaded gasoline, Pb levels in ambient air and placental tissue drastically decreased, and smoking-related differences became more apparent in subsequent periods. The concurrent decline in ambient air Pb and placental Pb provides strong biological evidence for the efficacy of regulatory actions in lowering maternal and fetal exposure. For Cd, the more gradual decline and the persistent smoker–nonsmoker gradient underscore the limitations of population-level environmental regulation when exposure is mainly driven by individual behaviour. Overall, these findings demonstrate that environmental regulations can effectively reduce exposure to metals originating from industrial or traffic sources, whereas ongoing and focused tobacco control efforts remain essential to further reduce Cd exposure.

Reducing placental Cd and Pb levels is especially important for toxicology and public health because the adverse effects of these metals during pregnancy are well established. Cd exposure has been associated with intrauterine growth restriction and lower birth weight [40,41] due to impaired placental function, oxidative stress, and disrupted transplacental transfer of essential elements, while exposure to Pb affects neurological development and increases the risk of preterm birth [42,43,44]. Both metals have been associated with oxidative stress [45], vascular problems [46], and epigenetic changes [47,48] that may influence fetal programming [49]. Maternal exposure to these toxic metals has also been associated with hypertension during pregnancy and impaired renal function [41,50]. In this context, biomonitoring of vulnerable groups, such as parturient women, using the placenta as a biological sample, provides an informative approach for evaluating prenatal exposure and its potential effects on maternal and fetal health.

To the best of our knowledge, this study provides the first comprehensive, long-term summary of placental Cd and Pb exposure in Croatia over a three-decade period, in a population that is methodologically and geographically comparable. By analyzing all available placental biomonitoring data from Zagreb maternity hospitals, this work offers a unique perspective on how major societal, environmental, and regulatory changes—such as the phase-out of leaded gasoline and the adoption of comprehensive tobacco-control measures—have led to measurable reductions in maternal and fetal metal exposure. Our findings also emphasize the importance of using the placenta as a biomonitoring matrix for assessing overall prenatal exposure and for identifying key gaps in the current database. They also highlight the need for continued surveillance as tobacco products and regulatory frameworks evolve.

This study has several limitations due to its design. First, the analysis relies on previously published studies and lacks individual-level data, preventing the use of formal statistical testing, adjustment for potential confounders (e.g., maternal age, parity, socioeconomic status), and harmonization of underlying data distributions across studies. Second, the limited number of available studies and small sample sizes, particularly for the 1990s, together with missing placental Pb data in two studies from 2008–2010, restrict the statistical power and robustness of comparisons across periods. Third, heterogeneity in how results were presented in the original studies (mean ± SD, median with interquartile range, or median with range) restricts quantitative comparability and prevents calculation of percentiles. Fourth, changes in analytical techniques over time may have introduced additional variability, despite broadly similar sampling procedures. Finally, restricting the dataset to Zagreb improved internal comparability due to geographic and population similarities but limited the generalizability of the findings to other regions of Croatia. Despite these limitations, the consistent pattern of observed trends over time across different studies provides strong evidence of a genuine temporal decline in Cd and Pb levels.

## 4. Conclusions

The assessment of Cd and Pb levels in placental tissue of women from the Zagreb area, Croatia, over time, based on selected studies conducted in Zagreb, Croatia, shows a significant decline in the amounts of both toxic metals over the past three decades (1990s–2019). This decline closely aligns with major public health initiatives, including implementation of the WHO Framework Convention on Tobacco Control (FCTC), reductions in smoking rates among women, increased public awareness of smoking-related risks, and improvements in educational structure. In parallel, the pronounced decrease of Pb in ambient air following the phase-out of leaded gasoline has also contributed significantly to the reduction of placental Pb levels. Overall, the combination of tobacco-control measures, including prohibition of smoking in public areas, improved health literacy in relation to smoking, and the transition to unleaded gasoline appears to have played a key role in the gradual decline of placental metal burden. However, the emergence and increasing popularity of heated and other novel tobacco products raise new public health concerns. Future research should therefore focus on elucidating the potential toxicological effects of these products on pregnant women and fetal health.

## Figures and Tables

**Table 1:** Summary of Cd and Pb concentrations in placentas of smoking and nonsmoking women from the Zagreb area, Croatia (1990–2019), based on published data *.

	Period	n	Cd (ng/g wet wt)	Pb (ng/g wet wt)	Sources
Nonsmokers	1990s	27	15.9 ± 1.44	49.5 ± 7.47	[17]
	1990s	109	9.70 (7.30–12.5)	13.8 (7.6- 27.1)	[18]
	2008–2010	119	6.48 (5.09–8.62)	/	[13]
	2008–2010	54	7.63 (3.34–18.8)	/	[19]
	2008–2010	156	7.6 (5.4–11)	5.4 (3.6–7.8)	[20]
	2017–2019	37	6.33 (5.45–8.59)	2.07 (1.62–2.99)	[21]
	2017–2019	37	5.48 (4.36–7.34)	1.44 (1.06–2.52)	[22]
	2017–2019	71	5.4 (4.3–7.7)	1.6 (1.1–2.5)	[23]
Smokers	1990s	25	28.5 ± 2.05	41.7 ± 6.87	[17]
	1990s	99	21.4 (17.2–26.5)	17.9 (10.3–34.2)	[18]
	2008–2010	77	10.5 (8.54–14.1)	/	[13]
	2008–2010	32	10.4 (5.42–33.6)	/	[19]
	2008–2010	112	11 (8.5–15)	6.1 (4.4–8.9)	[20]
	2017–2019	37	8.07 (6.03–11.9)	2.86 (2.24–5.07)	[21]
	2017–2019	35	7.62 (5.28–10.5)	2.56 (1.86–3.66)	[22]
	2017–2019	36	7.9 (5.6–12)	2.5 (1.8–3.5)	[23]

* Descriptive statistics are presented as reported in the original publications. The first two publications [17,18] did not specify the exact years of sample collection; accordingly, these data are broadly referred to as the 1990s.

## Data Availability

No new data were created or analyzed in this study.
